# Prevalence and characteristics of post-acute sequelae of COVID-19 in recovered patients

**DOI:** 10.3389/fpubh.2025.1648961

**Published:** 2025-09-18

**Authors:** Zuri Dale, Sherrie Flynt Wallington, Michelle Penn-Marshall

**Affiliations:** ^1^Texas Southern University, Houston, TX, United States; ^2^School of Nursing, George Washington University, Ashburn, VA, United States; ^3^Milken Institue School of Public Health, Washington, DC, United States; ^4^GW Cancer Center, Washington, DC, United States

**Keywords:** long COVID, post-acute sequelae of SARS-cov-2 (PASC), symptom severity, comorbidities, risk factors

## Abstract

**Introduction:**

Long COVID, also known as post-acute sequelae of SARS-CoV-2 infection, has emerged as a major public health concern following the COVID-19 pandemic. Although initially perceived as a respiratory illness, growing biomedical evidence confirms that COVID-19 affects multiple organ systems. This study aimed to explore the clinical manifestations, risk factors, and long-term outcomes associated with long COVID and to identify patients at highest risk. The research also contributes to the ongoing discourse on establishing a unified definition of long COVID.

**Methods:**

A secondary analysis of a cross-sectional, community-based study was conducted using data from 168 households, representing a weighted total of 14,769 households in Third Ward, Houston, Texas. Data were collected via interviewer-administered surveys and included variables on demographics, pre-existing comorbidities, COVID-19 symptom severity, and post-acute symptom persistence. Symptom variables were recoded as binary indicators, and weighted logistic regression models were applied to identify associations between acute phase characteristics and the development of long COVID.

**Results:**

Risk factors significantly associated with long COVID included symptom severity during acute infection (OR = 29.58, 95% CI [1.38, 632.53]), heart disease (OR = 6.00, 95% CI [1.15, 31.28]), asthma (OR = 3.49, 95% CI [1.05, 11.59]), and poor physical health (OR = 4.20, 95% CI [1.12, 15.75]). Acute symptoms predictive of long COVID included anxiety (OR = 22.94, 95% CI [2.01, 262.31]), chest pain (OR = 7.15, 95% CI [1.13, 45.23]), constipation (OR = 16.81, 95% CI [1.33, 213.23]), heart palpitations (OR = 6.59, 95% CI [1.08, 40.18]), and shortness of breath (OR = 4.97, 95% CI [1.16, 21.36]). No statistically significant associations were found between long COVID and race, education, or income.

**Conclusion:**

The findings underscore the multisystemic nature of long COVID, characterized by a diverse range of symptoms including fatigue, cognitive impairment, shortness of breath, and neuropsychiatric issues such as depression. While clinical factors are critical in understanding long COVID, the results also suggest that addressing associated health outcomes requires broader consideration of social determinants of health.

## 1 Introduction and background

Globally, over 651 million individuals have confirmed cases of COVID-19, including 86 million in the United States (1). In April 2021, the U. S. Food and Drug Administration approved multiple vaccines for coronavirus, and despite highly transmissible variants like Omicron, COVID-19 vaccines are, in general, highly efficient in protecting against severe disease. Since the rollout of the COVID-19 vaccines, the number of COVID-19 survivors has exponentially increased. However, the unknown threat of post-COVID manifestations remains (2).

Although the severe course of the disease has been a concern since the early stages of the pandemic, post-recovery manifestations are now of increased concern (3). As of 2024, new impacts of the virus are still being identified, highlighting the ongoing uncertainty and the urgent need for further research. Emerging evidence indicates that patients may continue to suffer from persistent post-infectious symptoms (e.g., fatigue, brain fog, chest or throat pain, or dyspnea) for more than 2 months (median 72 days) and may also have at least one unscheduled outpatient visit up to 6 months post-diagnosis (4). As more information about patient recovery is collected, the range of outcomes following acute COVID-19 continues to grow.

Experiencing symptoms post-acute infection is not unexpected and has been characteristic of other infectious diseases like Lyme disease, Ross River Virus, Epstein–Barr virus, and coronaviruses like Severe Acute Respiratory Syndrome (5). SARS, caused by SARS-CoV, has been observed to cause persistent and new-onset symptoms at follow-ups conducted 15 years post-infection. For those infected with SARS-CoV, reported effects on the respiratory system (lung capacity and health), physiological health, bone health, and metabolism have been reported, with improvements happening within the first one to two years. However, decreased quality of life has been observed in a subset of patients over a decade after acute infection.

Post-COVID-19 condition (also known as long COVID) is generally defined as symptoms persisting for 3 months or more after acute COVID-19, however the nomenclature for defining the ongoing symptoms experienced has differed among institutions and within published literature (6). The Centers for Disease Control and Prevention (CDC) has formerly described the COVID-19-related symptoms that last longer than 4 weeks as “Post-Acute Sequelae of COVID-19 (PASC)” or “long COVID” (7). The World Health Organization (WHO) further defines long-term as the illness that occurs in people with a history of probable or confirmed SARS-CoV-2 infection, usually within 3 months (12 weeks) from the onset of COVID-19 with symptoms that last for at least 2 months (8). The National Institute of Health and Care Excellence (NICE) recommends using the term long COVID for signs and symptoms that occur more than 4 weeks and post-COVID syndrome for symptoms lasting more than 12 weeks after infection (9). Neither definition requires a positive laboratory test. In this study, the terms “Post-Acute Sequelae of COVID-19 (PASC)” or “long COVID” are used interchangeably to describe symptoms lasting longer than 4 weeks. Our definition of long COVID was taken together by a combination of those experiencing symptoms at least 30 days (4 weeks) beyond infection and having been diagnosed at least 12 weeks (3 months) prior to the survey (Table 1), however since the time of this data collection the National Academies of Medicine has also established a definition of long-COVID.

**Table 1:** Nomenclature for long COVID among institutions.

CDC	WHO	NICE	Current study
Symptoms that last longer than 4 weeks as “Post-Acute Sequelae of COVID-19” or “long COVID” (7)	Illness that occurs in people with a history of probable or confirmed SARS-CoV-2 infection, usually within 3 months (12 weeks) from the onset of COVID-19 with symptoms that last for at least 2 months (8)	Signs and symptoms that occur more than 4 weeks and post-COVID syndrome for symptoms lasting more than 12 weeks after infection (9)	Our definition of long COVID was based on a combination of those experiencing symptoms at least 30 days (4 weeks) beyond infection *and* having been diagnosed at least 12 weeks (3 months) prior to the survey.

Post-Acute Sequelae of COVID-19 (PASC) and long COVID are used interchangeably.

CDC=Centers for Disease Control and Prevention; WHO=World Health Organization; NICE = National Institute of Health and Care Excellence.

While it was initially thought that COVID-19 was only respiratory in nature, numerous biomedical reports have shown that it has had a multisystemic adverse impact on almost all systems, including the respiratory, cardiovascular, psychiatric, gastrointestinal, dermatological, musculoskeletal, nervous, and metabolic systems (Table 2). The delayed realization that COVID-19 did not only have respiratory sequelae has led to a disproportionate focus on respiratory rehabilitation and skewed electronic health record data. On the heels of the pandemic itself, long COVID has the potential to create another public health crisis.

**Table 2:** Long COVID symptoms and the impacts on numerous organs with differing pathology.

Symptoms	Pathology
Heart	
Chest Pain Palpitations	Cardiac impairment Myocardial inflammation POTS
Lungs	
Cough Dyspnoea	Abnormal gas exchange
Pancreas	
N/A	Diabetes Pancreas Injury
Gastrointestinal tract	
Abdominal pain Nausea	Gut dysbiosis Viral persistence and viral reservoir
Neurological System	
Cognitive Impairment Fatigue Disordered Sleep Memory Loss Tinnitus	Dysautonomia ME/CFS Neuroinflammation Reduced cerebral blood flow Small fibre neuropathy
Kidneys, Spleen, and Liver	
N/A	Organ Injury
Blood Vessels	
Fatigue	Coagulopathy Deep vein thrombosis Endothelial dysfunction Microangiopathy Microclots Pulmonary Embolism Stoke
Reproductive system	
Erectile dysfunction Increased severity and number of premenstrual symptoms Irregular Menstruation	Reduced sperm count

The impact of COVID-19 on multiple organ symptoms has presented management challenges. Table adapted and reproduced (public domain) from Davis et al. (1).

Based upon a conservative estimated incidence of 10%, it is assumed that at least 65 million individuals worldwide have long COVID-19, with undocumented cases likely resulting in a gross underestimate of the actual disease burden (1). Further contributing to data limitations is that it was not until October 2021 that a specific International Classification of Diseases Tenth Edition (ICD-10) diagnosis code for “post-COVID” conditions was determined. Despite this, between October 2021 and January 2022, only 78,252 privately insured patients were formally diagnosed with the U09.9 code for COVID-19 after acute infection (10).

Long COVID has been associated with a wide range of disease severities, female sex, high viral load, and a high percentage of diagnosis between ages 36 and 50 in non-hospitalized patients (10). The misconception that individuals who experienced mild or no symptoms during acute infection would not have long-term consequences has had downstream effects on long COVID treatment and management (3). As a result, these patients have not been as widely studied as hospitalized patients. The lack of a validated effective treatment for long COVID and the fact that it can occur irrespective of the severity of the initial infection underscores the urgency for finding a solution to the emerging disease (11). This is further compounded by the exact mechanisms that underlie long COVID being largely unknown.

Accessing adequate resources, support, medical assessment, and treatment for long COVID has been a challenge, particularly for those with no laboratory evidence of their infection. During the earlier onset of the pandemic, it is thought that only 1–3% of cases before March 2020 were detected due to testing being less readily available to those who were not hospitalized, undiagnoses, and inadequate assessmend of symptoms (1, 6, 12). In the United States, the CDC estimates that only 25% of cases were reported from February 2020 to September 2021. Most studies to date have focused on individuals previously hospitalized with COVID, with the assumption that hospitalization indicates severe disease in most settings. Individuals who experienced non-severe infection have been less studied. Therefore, the prevalence of long COVID in those who seemingly recovered from COVID-19 may help inform the need for rehabilitation and further investigation as long COVID may be a predictor of the future incidence and or exacerbation of chronic disease (13).

The natural history of COVID-19 does appear to improve gradually over time in most cases. However, some patients require a comprehensive assessment to exclude serious complications underlying their symptoms. In contrast, others who survived hospitalization and ICU admission and those with preexisting conditions may require more specialized care. Given the paucity of evidence, it is still challenging to determine which symptoms and issues related to long COVID are caused by the disease itself and which may be unrelated but more difficult to treat due to COVID and its after-effects. To further compound these challenges, COVID-19 and its long-term sequelae are not only influenced by clinical health factors but by non-clinical determinants such as race, income, education, access to healthcare, and structural inequalities of racism and discrimination (14).

Caring for persistent symptoms of COVID-19 increases hospitalization and financial burden for patients and the healthcare system (15). Moreover, vulnerable populations and those of moderate means may not seek necessary care (16). With significant proportions of individuals with long COVID unable to return to work, the increased number of newly disabled individuals is contributing to labor shortages, job security is decreased, and there is reduced availability of occupational health services. This underscores the need to assess and address the social needs of those with long COVID.

**Figure 1:**
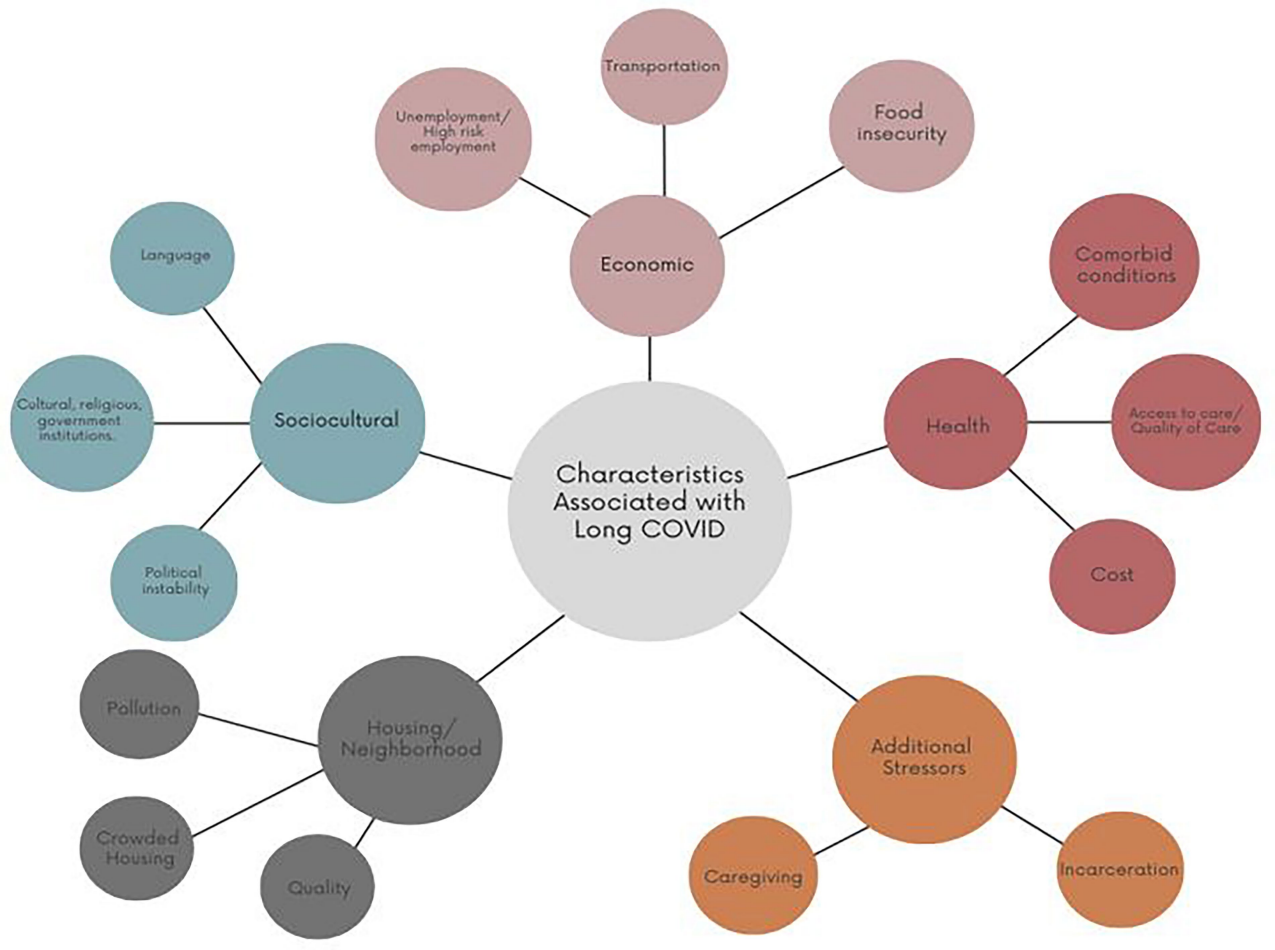
Characteristics associated with the development of long COVID. Adapted for use from Berger et al. (14).

While some risk factors, such as immunosuppression, are not modifiable, others, like the social determinants of health may be. As such, addressing clinical health factors also requires exploring the socioeconomic inequities underpinning them. In many societies’ income, education, occupation, and race/ethnicity are proxies for socioeconomic position (17). Therefore, race, lower incomes, lower education rates, and sociodemographic disadvantages of lack of health care are also expected to contribute to a subsequent risk of long-term COVID-19. The cumulative effect of COVID-19, non-communicable diseases associated with adverse outcomes, and the socioeconomic factors that contribute to non-communicable disease prevalence can be considered a syndemic (18). The relationship between structural inequalities and COVID-19 is illustrated in Figure 1, which presents a conceptual framework outlining key characteristics associated with long COVID. These include health-related vulnerabilities (e.g., comorbid conditions, limited access to care), economic challenges (e.g., unemployment, food insecurity), sociocultural dimensions (e.g., language barriers, political instability), housing and neighborhood factors, and psychosocial stressors such as caregiving burdens. These interconnected domains provide important context for understanding the complex patterns of symptom persistence and recovery trajectories following COVID-19.

Most existing literature focuses on the frequency of individual symptoms instead of population-based estimations. Moreover, there is a need for the simultaneous consideration of multiple persistent symptoms and comorbidities over time. The objective of this study is to more fully (1) understand and characterize the spectrum of post-acute sequelae of COVID-19 (2) identify individuals who are most susceptible to the development of post-acute sequelae of COVID-19 and (3) contribute to gaps in understanding epidemiological, clinical, and nonclinical risk factors that contribute to post-acute sequelae of COVID-19. This study aims to evaluate the association between clinical course of disease severity, comorbidities, race, income, education, and healthcare access and the development of post-acute sequelae of COVID-19 in patients seemingly recovered from COVID-19.

Understanding these associations has important public health implications. It supports the prioritization of preventive measures, vaccination, and early treatment, while also offering insights into the biological mechanisms of long COVID (19). Public health guidance already reflects these priorities, emphasizing protection for high-risk groups. Investigating risk factors in disproportionately affected communities can reveal barriers to recovery and inform targeted interventions. Moreover, engaging communities of color in research and clinical trials ensures more equitable outcomes and improves the relevance and impact of findings. Lived experience is essential to shaping effective study design and accelerating progress (1).

## 2 Methodology

### 2.1 Study design

CASPER, a cross-sectional two-stage cluster sampling methodology, was utilized to collect the data that underwent secondary analysis. CASPER is a validated method developed for rapid needs assessment by the Centers for Disease Control and Prevention (CDC) to rapidly obtain information about the needs of a community (20). While it was originally designed for emergency management disaster settings, there are opportunities for CASPER to influence public health in non-disaster settings as CASPER has been previously used to estimate community needs and assess public health perceptions. In using data from the CASPER methodology in this study, household-based population estimates are determined to ensure the sample is generalizable to the larger population. By design, CASPER instruments collect cross-sectional data about the entire household with data obtained from an individual household member. All questions analyzed were asked at the household level.

### 2.2 Sample population and size

The CASPER was conducted in the Third Ward in Houston, Texas. The Third Ward has a resident population of 38, 920 and has seen a population increase of 1.49 percent since 2020. The population by race is Black/African American (38.43%), White (30.75%), American Indian (0.56%), Asian (14.01%), Native Hawaiian/Pacific Islander (0.05%), Other (7.46%) and/or Mixed (8.74%). The median household income is $59,026 with 18.72% of families living below the poverty line ($23,400) for a family of four. One hundred sixty-eight unique households completed the survey instrument, representing a weighted population size of 14, 769 total households. Exploring the baseline health in Third Ward was a cornerstone of this study and was needed to explore associations between comorbid conditions and long COVID. The prevalence of disease in Third Ward prior to COVID-19 was higher than national rates for many of the health indicators selected in this study.

### 2.3 Study analytic plan

The data used for secondary analysis was collected by an instrument that included information regarding household demographics (age, income, education, race/ethnicity, employment status), prevalence of chronic conditions (heart disease. Stroke, asthma, cancer, cholesterol, obesity, diabetes, depression, anxiety, stroke, COPD, and chronic kidney disease) and post-COVID impact (history of hospitalization, length of time since diagnosis, symptom severity, presence of persistent symptoms, outpatient visits, impact on activities of daily living).

One hundred sixty-eight unique households completed the interviewer-administered survey. Each household for which an interview was completed was assigned a weight based on the household’s probability of selection. This weighting ensured that the resulting estimates were generalizable to all household in the sampling frame, with each cluster weighted equally. SAS 9.4 was utilized for the analysis allowing for appropriate multistage sample design weighting. The following weight formula was used: Weighted frequencies, percentages, and 95% confidence intervals were then calculated for each of the interview questions for cells with five or more observations. A total of 14, 769 households were included in the 77,004-sampling frame and used in the weight calculation. Once weight was assigned, frequencies and corresponding percentages were calculated for each question.

### 2.4 Univariate and bivariate analysis

Descriptive assessments were done among the full cohort, but research questions (inferential statistics) were only conducted and among weighted households (*N* = 7,088) with members who tested positive for COVID or had symptoms. Participants who reported not having any COVID symptoms were then further excluded from the inferential analyses, bringing the study cohort for the associations between risk factors and long COVID to 6,418 weighted households.

Bivariate (crude) and multivariable logistic regression models were constructed to determine associations between risk factors and long COVID. Five separate logistic regression models were constructed with unadjusted and adjusted odds ratios (OR) and 95% confidence intervals (CI) reported for each risk factor, where applicable. The study outcome was a binary measurement of the development of post-acute sequalae of COVID-19. The variable long COVID was created together by a combination of those experiencing symptoms at least 30 days beyond infection and having been diagnosed at least 12 weeks (3 months) prior to the survey. Odds ratios were obtained, and confidence intervals were used to determine whether statistical significance had been reached.

### 2.5 Cleaning of imported data and variable recoding

Due to limited data, some variables (household income and educational level) required recoding. In the original survey instrument, respondents were asked to “check all that apply” to indicate the presence of COVID-19 symptoms. For analysis, each symptom variable was separated, and a binary yes/no response was created.

### 2.6 Statistical methods for the study outcomes

Potential risk factors for COVID-19 were identified using existing literature, clinical expertise and the Charlson Comorbidity Index, supplemented by additional comorbidities documented in prior studies (21). Research questions, variable measurement levels, and corresponding statistical analyses are summarized in Table 3.

**Table 3:** Research questions and variables by level of measurement and statistical analysis performed.

Research question	Predictors	Outcome	Analysis performed
RQ 1	Symptom severity (categorical)	Long COVID (binary)	Bivariate and Multivariate logistic regression models
	Mild (Reference)		
	Moderate		
	Severe		
RQ 2	Existing conditions (categorical)	Long COVID (binary)	Bivariate and Multivariate logistic regression models
	Heart Disease		
	High Blood Pressure		
	Stroke		
	Asthma		
	Cancer		
	High Cholesterol		
	Overweight/Obesity		
	Diabetes/High Blood Sugar		
	Poor Mental Health		
	Poor Physical Health		
	COPD		
	Chronic Kidney Disease		
RQ 3	Race/ethnicity (categorical)	Long COVID (binary)	Bivariate and Multivariate logistic regression models
	Hispanic		
	Non-Hispanic Black/African American		
	Non-Hispanic Asian, Native Hawaiian or Pacific Islander, or Other		
	Non-Hispanic White (Reference)		
RQ 4	Income (categorical)	Long COVID (binary)	Bivariate and Multivariate logistic regression models
	< $25,000		
	$25–000-$49,000		
	$50,000–$74,000		
RQ 5	Highest education level (categorical)	Long COVID (binary)	Bivariate and Multivariate logistic regression models
	High school or less		
	College or more		
RQ 6	Access to healthcare (categorical)	Long COVID (binary)	Bivariate and Multivariate logistic regression models
	No		
	Yes		

## 3 Results

### 3.1 Demographic characteristics of the population

The final descriptive analysis includes 168 households in Third Ward representing 14,769 weighted households. Weighted frequencies, percentages, and 95% confidence intervals were then calculated for each of the interview questions for cells with five or more observations. Of the participants, 57.78% (*n* = 8,432) of households identified as Non-Hispanic Black/African American and 16. 78% (*n* = 2,449) identified as Hispanic. The majority of households identified as employed (42.45%, *n* = 6,076), had a household income of $75,000 or more (29.70%, *n* = 3,999), and had at least one individual in the home who had attended college (21.94%, *n* = 9,938). Most respondents identified the members of their household as representing the 18–64 age group (73.35%, *n* = 10,833). Complete descriptive characteristics of the surveyed population are shown in Table 4.

**Table 4:** Weighted and unweighted frequencies of descriptive characteristics for households in third ward Houston, TX.

Descriptive characteristics	Unweighted *N* (168)	Weighted *N*	Weighted % HH	95% CI (lb)	95% CI (ub)
Type of structure					
Single Family Homes	108	9,820	66.89	54.74	79.05
Multiple Units	57	4,684	31.9	19.28	44.52
Other	2	177	1.2	0	2.92
Missing	1	–	–	–	–
Primary language					
English	11	12,825	92.51	86.80	98.22
Spanish	2	862	6.22	0.54	11.89
Other	12	177	1.27	0.00	3.10
Missing		–	–	–	–
Annual income					
Less than $10,000	33	2,430	18.05	7.42	28.68
Less than $25,000	12	965	7.16	1.83	12.50
Less than $35,000	9	766	5.69	1.43	9.95
Less than $50,000	29	2,408	17.88	9.82	25.95
Less than $75,000	34	2,898	21.52	12.43	30.61
$75,000 or more	36	3,999	29.70	16.20	43.19
Missing	15	–	–	–	–
Highest education level					
Never Attended School	2	177	1.33	0.00	3.25
Elementary School	1	44	0.3	0.00	1.01
Middle School	3	243	1.82	0.00	4.00
High School	38	2,924	21.94	13.67	30.21
College	104	9,938	74.58	65.93	83.22
Missing	151	–	–	–	–
Employment status					
Retired	29	2,607	18.21	10.86	25.57
Self-Employed	36	3,030	21.17	14.46	27.88
Student	15	1,296	9.06	0.00	19.12
Unable to Work	7	449.22	3.14	0.37	5.91
Unemployed	10	854.26	5.97	1.16	10.78
Employed	66	6,076	42.45	31.27	53.62
Missing	5	–	–	–	–
Race					
Hispanic	30	2,449	16.78	8.17	25.39
Non-Hispanic Black/African American					
Hispanic	103	8,432	57.78	42.7	72.78
Non-Hispanic Asian, Native Hawaiian or Pacific Islander, or other	7	608	4.16	1.34	6.99
Non-Hispanic White	26	3,104	21.27	8.18	34.36
Missing	2	–	–	–	–
Age (years)					
<2					
No	163	14,408	97.56	95.41	99.71
Yes	5	361	2.44	0.29	4.59
2–17					
No	140	12,494	84.59	76.94	92.25
Yes	28	2,276	15.41	7.75	23.06
18–64					
No	47	3,936	26.65	16.36	36.95
Yes	121	10,833	73.35	63.05	83.64
≥65					
No	125	11,275	76.34	66.69	85.99
Yes	43	3,494	23.66	14.01	33.31

*n* = Frequency of households (HH).

CI (ub) = Confidence interval upper bound.

CI (lb) = Confidence interval lower bound.

### 3.2 Clinical characteristics of population

**Figure 2:**
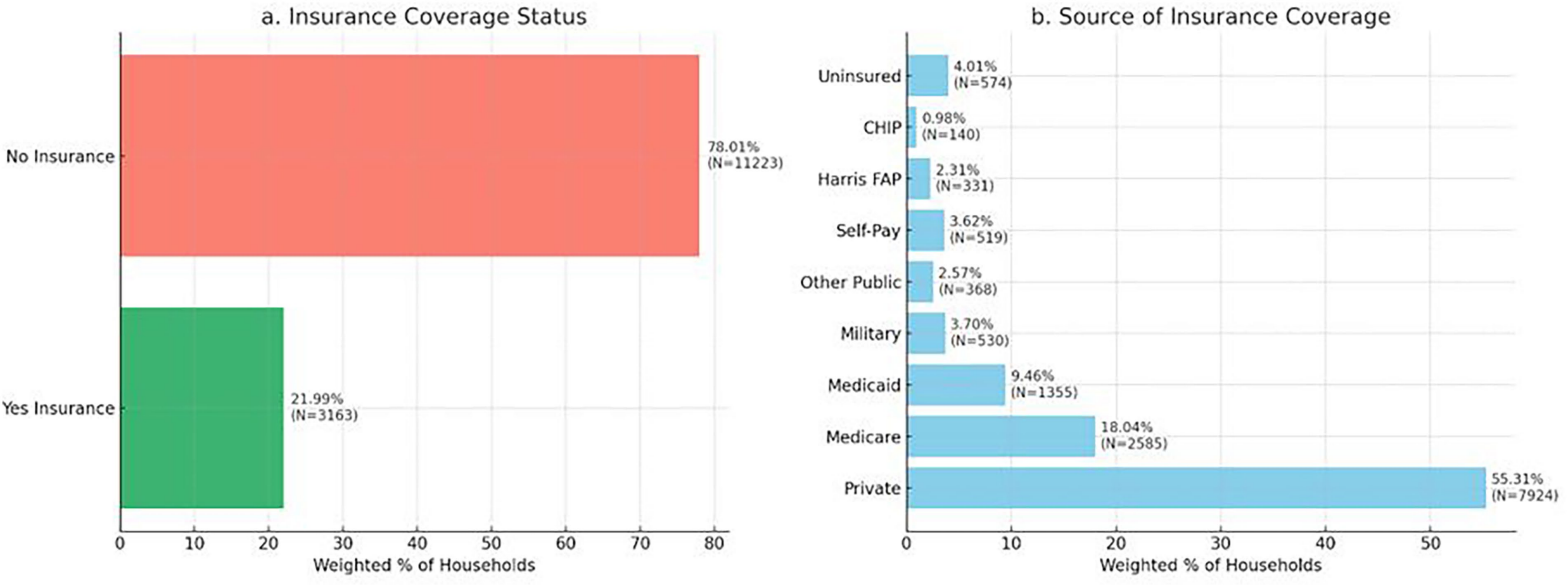
Insurance coverage status and type among households in the third ward, Houston, TX.

Approximately 70 % of households (78.01%, *n* = 11,233) of households identified as not having health insurance coverage and cited a doctor’s office as the place visited most often to see a doctor (52.81%, *n* = 7,707). 83.86%, *n* = 12,011 of households indicated no difficulty in getting medical services in the prior 12 months. Of those who cited difficulty (16.14%, *n* = 2,312), lack of insurance represented the greatest barrier to care (39.33%, *n* = 950). The clinical characteristics of the study population are shown in Figure 2 and Table 5.

**Table 5:** Weighted and unweighted frequencies of healthcare access characteristics for households in the third ward Houston, TX.

Healthcare access characteristic	Unweighted *N*	Weighted *N*	Weighted % HH	95% CI (lb)	95% CI (ub)
Access to personal or family doctor					
No	28	2,894	20.67	9.02	32.31
Yes	130	11,109	79.33	67.69	90.98
Missing	10	–	–	–	–
Difficulty getting medical services					
No	135	12,011	83.86	77.24	90.47
Yes	28	2,312	16.14	9.53	22.76
Missing	5	–	–	–	–
Place visited most often to see a doctor					
A clinic of health center	57	5,199	35.63	22.97	48.29
A doctor’s office or other provider office	88	7,707	52.81	39.03	66.59
A hospital emergency room	9	707	4.84	1.35	8.34
A hospital outpatient department	10	803	5.50	1.61	9.40
Some other place	2	177	1.21	0.00	2.94
Missing	2	–	–	–	–
Reason for difficulty getting medical services					
Do not have a car or transportation	3	280	11.59	0.00	23.61
Do not have a doctor/clinic	4	353	14.63	0.00	29.83
Do not have enough money to pay for health care	6	464	19.21	4.14	34.28
Do not have insurance	11	950	39.33	20.30	58.35
Other reasons	5	368	15.24	2.64	27.85
Yes	139				

*n* = Frequency of households (HH).

CI (ub) = Confidence interval upper bound.

CI (lb) = Confidence interval lower bound.

### 3.3 Medical characteristics and comorbid conditions

**Figure 3:**
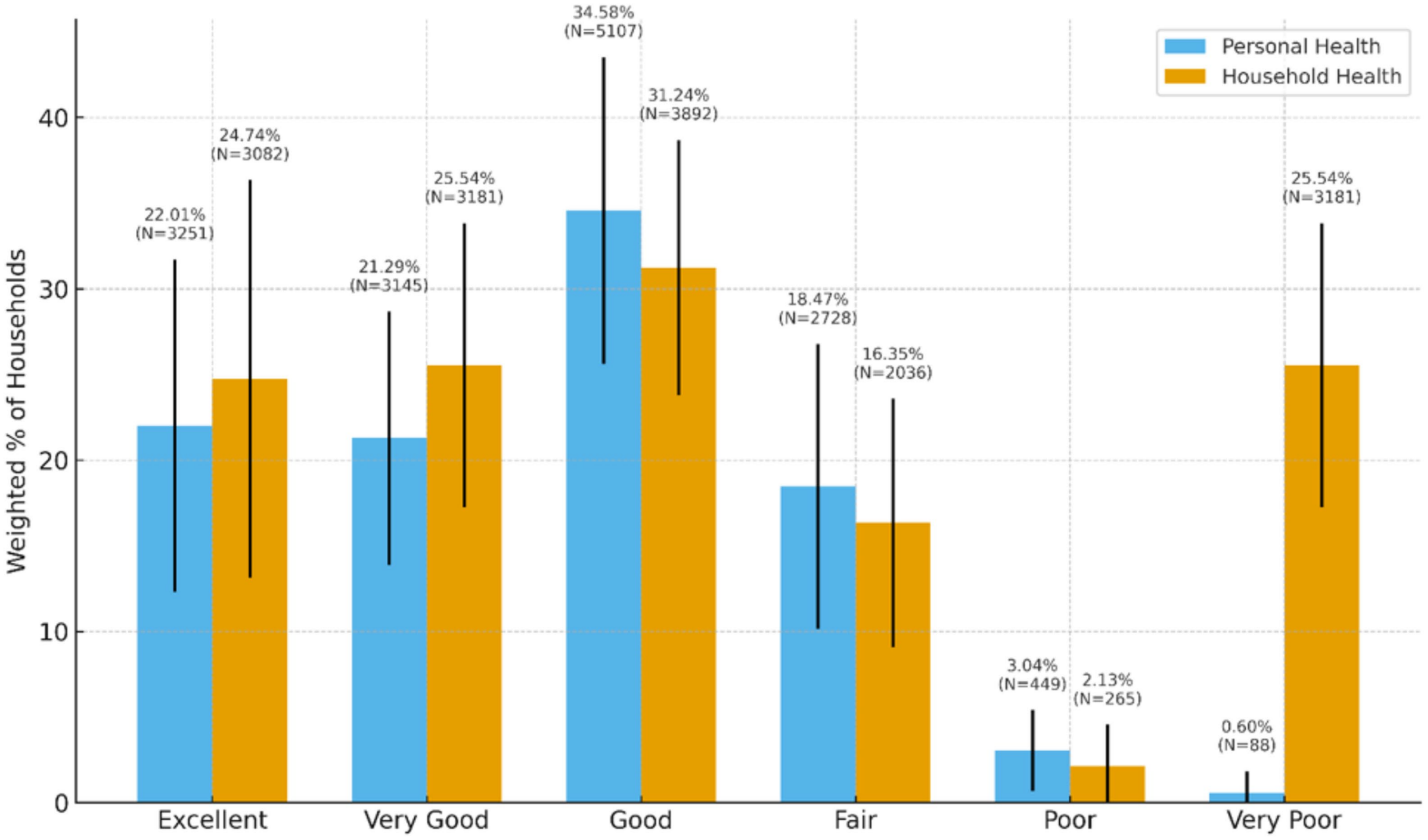
Weighted distribution of self-rated personal and household health status among surveyed households.

Approximately 81.5% of households rated their health as excellent (24.74%, *N* = 3,082), very good (25.54%, *N* = 3,181), or good (31.24%, *N* = 3,892). Figure 3 presents the weighted distribution of self-rated personal and household health status among surveyed households. High blood pressure (36.79%, *n* = 5,321), high cholesterol (25.92%, *n* = 3,701), poor mental health (19.18%, *n* = 2,740), obesity (18.79%, *n* = 2,699) and diabetes/high blood sugar (17.53%, *n* = 2,511) were cited as the more prevalent health conditions. Weighted and unweighted frequencies of health status/well-being for households can be found in Table 6.

**Table 6:** Weighted and unweighted frequencies of health status/well-being for households in third ward Houston, TX.

Health status	Unweighted *N* = 168	Weighted *N*	Weighted % HH	95% CI (lb)	95% CI (ub)
Heart DISEASE					
No	139	12,398	87.52	81.57	93.48
Yes	21	1767	12.48	6.52	18.43
Missing	8	–	–	–	–
Blood pressure					
No	103	9,143	63.21	53.26	73.16
Yes	61	5,321	36.79	26.84	46.74
Missing	4	–	–	–	–
Stroke					
No	152	13,388	93.14	89.03	97.24
Yes	11	987	6.86	2.76	10.97
Missing	5	–	–	–	–
Asthma					
No	139	12,383	87.15	81.4	92.9
Yes	22	1826	12.85	7.1	18.6
Missing	7	–	–	–	–
Cancer					
No	145	12,611	89.38	82.94	95.82
Yes	15	1,499	10.62	4.18	17.06
Missing	8	–	–	–	–
High cholesterol					
No	116	10,579	74.08	67.24	80.93
Yes	45	3,701	25.92	19.07	32.76
Missing	7				
Overweight/obesity					
No	131	11,665	81.21	74.32	88.1
Yes	32	2,699	18.79	11.9	25.68
Missing	5	–	–	–	–
Diabetes/high blood sugar					
No	132	11,812	82.47	75.75	89.18
Yes	30	2,511	17.53	10.82	24.25
Missing	6	–	–	–	–
Poor mental health					
No	133	11,547	80.82	72.53	89.12
Yes	29	2,740	19.18	10.88	27.47
Missing	6	–	–	–	–
Poor physical health					
No	141	12,567	87.96	82.29	93.64
Yes	21	1720	12.04	6.36	17.71
Missing	6	–	–	–	–
Chronic obstructive pulmonary disease					
No	151	13,388	94.89	91.26	98.51
Yes	9	722	5.11	1.49	8.74
Missing	8	–	–	–	–
Chronic kidney disease					
No	154	13,661	96.21	93.53	98.9
Yes	7	538	3.79	1.1	6.47
Missing	7	–	–	–	–

*n* = Frequency of households (HH).

CI (ub) = Confidence interval upper bound.

CI (lb) = Confidence interval lower bound.

### 3.4 COVID characteristics and post-COVID manifestations

**Figure 4:**
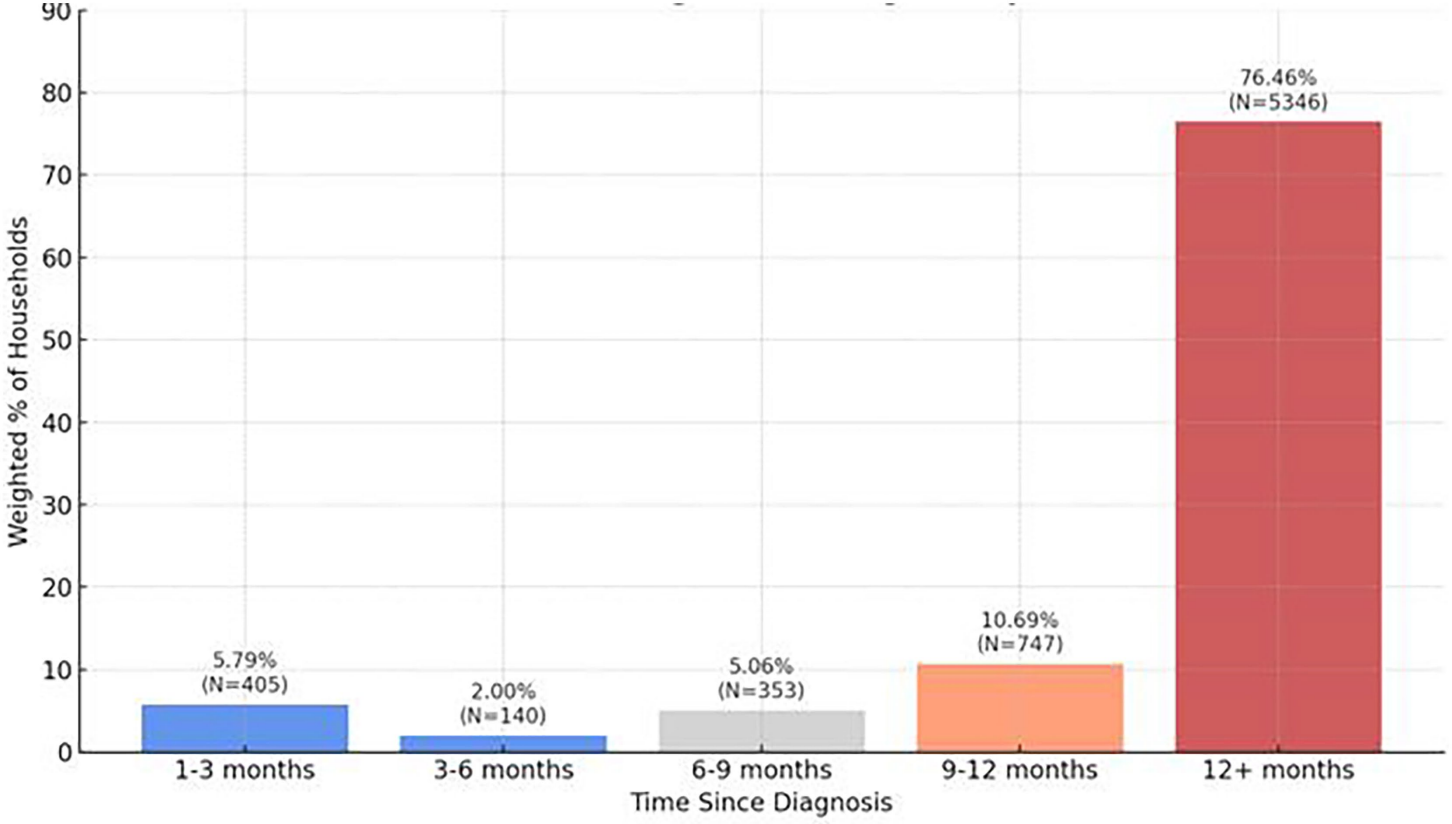
Distribution of time since COVID-19 diagnosis.

Approximately half of households (48.87%, *n* = 7,088) reported having tested positive for COVID-19 with the majority of households (76.46%, *n* = 5,346) reporting it had been 12 months or more since their diagnosis (Figure 4; Table 7). From Figure 4, most respondents had been diagnosed over 12 months ago, suggesting long-term persistence of symptoms and relevance to long COVID. 18.06%, *n* = 1,134 reported long COVID (still experiencing symptoms beyond 30 days after their initial COVID-19 infection). The majority of households reported moderate symptoms (50.77%, *n* = 3,505) with 12.05% (*n* = 832) of households indicating symptoms to be severe. Systemic symptoms including tiredness or fatigue, joint or muscle pain, taste or smell changes, headache, brain fog, and shortness of breath were the most frequently experienced household symptoms anytime during infection.

**Table 7:** Weighted and unweighted frequencies of COVID-19 characteristics and symptoms experienced during COVID-19 infection for households in third ward Houston, TX.

COVID characteristic and symptoms	Unweighted *N* = 168	Weighted *N*	Weighted % HH	95% CI (lb)	95% CI (ub)
Positive COVID test or symptoms					
No	91	7,416	51.13	41.05	61.21
Yes	74	7,088	48.87	38.79	58.95
Missing	3	–	–	–	–
Still experiencing COVID symptoms					
No	50	5,144	81.94	68.39	95.48
Yes	13	1,134	18.06	4.52	31.61
Missing	105	–	–	–	–
Time since diagnosis					
1 to 3 months	5	405	5.79	0.00	12.12
3 to 6 months	2	140	2.00	0.00	5.03
6 to 9 months	4	353	5.06	0.00	10.91
9–12 months	9	747	10.69	3.06	18.32
12 months or more	52	5,346	76.46	63.82	89.1
Long COVID					
No	50	5,144	81.94	68.39	95.48
Yes	13	1,134	18.06	4.52	31.61
Missing	105	–	–	–	–
Worst COVID symptoms					
No symptoms	3	486	7.04	0.00	16.17
Mild	25	2080	30.13	16.45	43.81
Moderate	32	3,505	50.77	36.37	65.18
Severe	11	832	12.05	4.81	19.3
Missing	97	–	–	–	–
Hospitalized for COVID					
No	61	6,028	87.77	79.24	96.31
Yes	10	840	12.23	3.69	20.76
Missing	97	–	–	–	–
Outpatient clinic visit after COVID recovery					
No	54	5,505	79.73	69.38	90.09
Yes	17	1,399	20.27	9.91	30.62
Missing	97	–	–	–	–

*n* = Frequency of households (HH).

CI (ub) = Confidence interval upper bound.

CI (lb) = Confidence interval lower bound.

**Figure 5:**
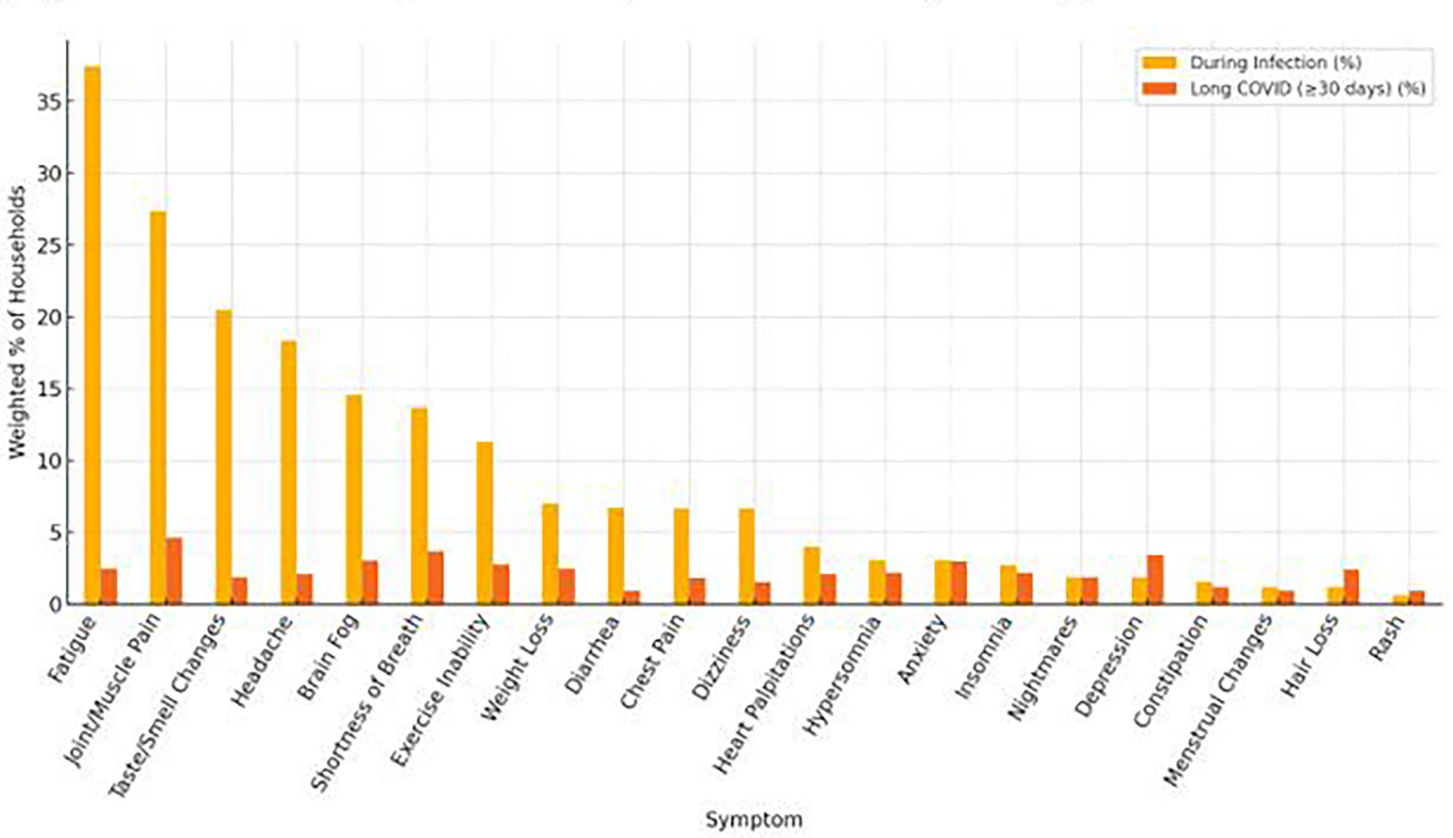
Symptom prevalence during COVID 19 infection and 30 + days post-infection.

The most persistent symptoms lasting 30 days or more included anxiety, brain fog, depression, joint or muscle pain, shortness of breath, exercise inability, tiredness or fatigue, and weight loss. To better illustrate the comparative burden of symptoms during acute infection and their persistence into the post-acute period, Figure 5 presents the weighted percentage of households reporting each symptom both during infection and 30 days or more afterward. This visualization reveals that while symptoms like fatigue (37.4%), joint or muscle pain (27.4%), and changes in taste or smell (20.5%) were highly prevalent during the acute phase, their persistence into the long COVID period declined substantially. In contrast, symptoms such as anxiety, brain fog, and depression, though less prevalent during acute illness, demonstrated notable persistence over time.

Eight hundred and forty (12.23%) households indicated at least one household member was hospitalized for COVID with 20.27% (*n* = 1,399) reporting at least one outpatient clinic visit after COVID recovery. 1,134 (18.06%) households reported long COVID (Table 7). We also explored activities of daily living impacted by COVID-19 symptoms, but given these characteristics fall outside of the scope of work comprehensive data was not reported. However, shopping, meal preparation, and household chores were cited to be among the activities of daily living most impacted.

### 3.5 Bivariate and multivariate analysis

This study sought to explore associations between select demographic, clinical, epidemiological, and non-clinical factors and long COVID. Both bivariate (crude) and multivariable logistic regression models were employed to determine these associations. The study outcome was a binary measurement of the development of post-acute sequalae of COVID-19. The variable long COVID was created together by a combination of those experiencing symptoms at least 30 days beyond infection and having been diagnosed at least 12 weeks (3 months) prior to the survey in accordance with the CDC definition for long COVID. Odds ratios (ORs) with corresponding confidence intervals were calculated to assess statistical significance. The complete results of the unadjusted and adjusted logistic regression models predicting long COVID are presented in Tables 8–10.

**Table 8:** Bivariate analysis for the association between characteristics of population and long COVID.

Crude			
Predictor	Odds Ratio (OR)	95% CI (ub)	95% CI (lb)
Severity of symptoms			
Mild	REF	–	–
Moderate	2.01	0.23	17.67
Severe	**14.40**	**1.59**	**130.27**
Race			
Hispanic	1.58	0.16	15.39
Non-Hispanic Black/African American	1.46	0.27	7.80
Non-Hispanic Asian, Native Hawaiian or Pacific Islander, or Other	1.90	0.10	36.88
Non–Hispanic White	REF	–	–
Annual HH Income			
Less than $25,000	1.70	0.19	15.04
$25,000 to $49,000	0.55	0.04	8.59
$50,000 to $74,000	0.87	0.19	4.00
$75,000 or more	REF	–	–
Highest education level			
High school or less	0.72	0.08	6.43
College or more	REF	–	–
65 years and older			
No	REF	–	–
Yes	2.13	0.36	12.62
Health insurance coverage			
No	0.54	0.17	1.76
Yes	REF	–	–
Heart disease			
No	REF	–	–
Yes	**3.97**	**1.12**	**14.02**
Blood pressure			
No	REF	–	–
Yes	1.57	0.37	6.71
Stroke			
No	REF	–	–
Yes	2.27	0.40	12.80
Asthma			
No	REF	–	–
Yes	**3.49**	**1.05**	**11.59**
Cancer			
No	REF	–	––
Yes	0.86	0.19	3.85
High cholesterol			
No	REF	–	––
Yes	3.73	0.77	18.2
Overweight or obesity			
No	REF	–	–
Yes	1.38	0.35	5.46
Diabetes or high blood sugar			
No	REF	–	–
Yes	3.63	0.82	16.06
Poor mental health			
No	REF	–	–
Yes	2.06	0.61	6.96
Poor physical health			
No	REF	–	–
Yes	**4.66**	**1.29**	**16.82**
COPD			
No	REF	–	–
Yes	4.32	0.28	67.07
Chronic kidney disease			
No	REF	–	–
Yes	3.54	0.52	24.21
COVID symptoms anytime during infection			
Anxiety			
No	REF	–	–
Yes	**17.26**	**1.55**	**192.54**
Brain fog			
No	REF	–	–
Yes	2.48	0.52	11.83
Chest pain			
No	REF	–	–
Yes	**6.93**	**2.02**	**23.77**
Constipation			
No	REF	–	–
Yes	**16.81**	**1.33**	**213.23**
Depression			
NULL			
Diarrhea			
No	REF	–	–
Yes	1.00	0.22	4.62
Dizziness	REF	–	–
No	1.00	0.22	4.62
Yes	REF	–	–
Exercise inability			
No	REF	–	–
Yes	2.70	0.70	10.47
Hair Loss			
NULL			
Heart palpitations			
No	REF	–	–
Yes	**6.76**	**1.74**	**26.34**
Hypersomnia			
No	REF	–	–
Yes	**25.34**	**2.36**	**272.05**
Insomnia			
No	REF	–	–
Yes	6.98	0.62	79.14
Joint or muscle pain			
No	REF	–	–
Yes	3.32	0.51	21.49
Menstrual changes			
NULL			
Nightmares			
NULL			
Rash			
NULL			
Shortness of breath			
No	REF	–	–
Yes	**4.38**	**1.11**	**17.3**
Taste or smell changes			
No	REF	–	–
Yes	0.98	0.30	3.23
Tiredness or fatigue			
NULL			
Weight loss			
No	REF	–	–
Yes	2.63	0.53	13.14
COVID symptoms lasting 30 days or more			
Anxiety			
No	REF	–	–
Yes	16.07	0.73	354.93
Brain Fog			
NULL			
Chest Pain			
NULL			
Constipation			
NULL			
Depression			
No	REF	–	–
Yes	4.02	0.69	23.32
Diarrhea			
No	REF	–	–
Yes	7.70	0.51	116.61
Dizziness			
No	REF	–	–
Yes	**14.08**	**2.30**	**86.06**
Exercise inability			
NULL			
Hair Loss			
NULL			
Heart palpitations			
NULL			
Hypersomnia			
No	REF	–	–
Yes	**21.62**	**1.78**	**262.93**
Insomnia			
NULL			
Joint or muscle pain			
No	REF	–	–
Yes	**19.89**	**2.63**	**150.69**
Menstrual changes			
NULL			
Nightmares			
NULL			
Rash			
No	REF	–	–
Yes	7.70	0.51	116.61
Shortness of breath			
No	REF	–	–
Yes	**25.34**	**3.87**	**165.75**
Taste or smell changes			
NULL			
Tiredness or fatigue			
NULL			
Weight loss			
No	REF	–	–
Yes	**27.91**	**3.97**	**196.07**

Bold = Significant association at alpha = 0.05.

NULL = Model failed to merge.

REF = Reference Group.

**Table 9:** Multivariate analysis for the association between characteristics of population and long COVID adjusted sequentially for age and age and race.

Predictor	Adjusted*			Adjusted**		
	Odds Ratio	95% CI (lb)	95% CI (ub)	Odds Ratio	95% CI (lb)	95% CI (ub)
Severity of symptoms						
Mild	REF	–	–	REF	–	–
Moderate	2.53	0.16	39.6	2.64	0.10	67.32
Severe	**15.88**	**1.16**	**216.99**	**16.21**	**1.09**	**241.28**
Race						
Hispanic	1.50	0.16	14.32	N/A	N/A	N/A
Non–Hispanic Black/African American	1.29	0.22	7.59	N/A	N/A	N/A
Non–Hispanic Asian, Native Hawaiian or Pacific Islander, or Other	2.09	0.11	40.75	N/A	N/A	N/A
Non–Hispanic White	REF	–	–	–	–	–
Annual HH income						
Less than $25,000	2.01	0.16	25.07	1.70	0.11	25.45
$25,000 to $49,000	0.62	0.03	12.4	0.65	0.02	27.18
$50,000 to $74,000	1.00	0.18	5.45	0.93	0.17	5.20
$75,000 or more	REF	–	–	REF	–	–
Highest Education Level						
High school or less	0.84	0.09	8.09	NULL		
College or more	REF	–	–	–	–	–
65 years and older						
No	REF	–	–	REF	–	–
Yes	N/A	N/A	N/A	2.15	0.27	17.17
Health insurance coverage						
No	0.53	0.17	1.60	0.48	0.13	1.73
Yes	REF	–	–	REF	–	–
Heart disease						
No	REF	–	–	REF	–	–
Yes	**3.52**	**1.05**	**11.8**	**3.45**	**1.05**	**11.35**
Blood pressure						
No	REF	–	–	REF	–	–
Yes	1.37	0.37	5.15	1.31	0.29	5.84
Stroke						
No	REF	–	–	REF	–	–
Yes	1.82	0.31	10.63	1.84	0.29	11.53
Severity of symptoms						
Asthma						
No	REF	–	–	REF	–	–
Yes	3.16	0.98	10.22	3.44	0.88	13.51
Cancer						
No	REF	–	–	REF	–	–
Yes	0.82	0.17	4.00	0.88	0.20	3.82
High cholesterol						
No	REF	–	–	REF	–	–
Yes	3.44	0.79	14.9	3.45	0.69	17.31
Overweight or obesity						
No	REF	–	–	REF	–	–
Yes	1.31	0.34	5.12	1.20	0.24	5.97
Diabetes or high blood sugar						
No	REF	–	–	REF	–	–
Yes	3.40	0.51	22.59	3.41	0.53	21.85
Poor mental health						
No	REF	–	–	REF	–	–
Yes	2.18	0.68	7.03	2.30	0.68	7.82
Poor physical health						
No	REF	–	–	REF	–	–
Yes	**4.20**	**1.12**	**15.75**	7.92	0.88	71.55
COPD						
No	REF	–	–	REF	–	–
Yes	3.38	0.09	121.13	3.68	0.11	129.16
Chronic kidney disease						
No	REF			REF		
Yes	3.10	0.34	28.08	2.91	0.30	28.31
COVID symptoms anytime during infection						
Anxiety						
No	REF	–	–	NULL		
Yes	**22.94**	**2.01**	**262.31**			
Brain Fog						
No	REF	–	–	REF	–	–
Yes	2.46	0.56	10.81	4.44	0.44	45.34
Chest Pain						
No	REF	–	–	REF	–	–
Yes	**6.85**	**2.08**	**22.53**	**7.45**	**1.76**	**31.55**
Constipation						
No	REF	–	–	REF	–	–
Yes	17.06	0.92	315.63	21.53	0.81	573.03
Depression	NULL					
Diarrhea						
No	REF	–	–	REF	–	–
Yes	1.03	0.23	4.69	1.15	0.24	5.63
Dizziness						
No	REF	–	–	REF	–	–
Yes	3.06	0.76	12.36	3.71	0.86	16.05
Exercise inability						
No	REF			REF		
Yes	2.48	0.65	9.52	2.64	0.64	10.93
Hair Loss						
NULL						
Headache						
No	REF	–	–	REF	–	–
Yes	1.42	0.41	4.94	1.62	0.47	5.59
Heart palpitations						
No	REF	–	–	REF	–	–
Yes	**6.14**	**1.42**	**26.63**	**6.84**	**1.73**	**27.14**
Hypersomnia						
No	REF	–	–	REF	–	–
Yes	**26.87**	**2.62**	**275.38**	**37.14**	**3.02**	**456.47**
Insomnia						
No	REF	–	–	REF	–	–
Yes	7.11	0.68	74.42	7.41	0.55	99.01
Joint or muscle pain						
No	REF			REF		
Yes	4.07	0.62	26.82	4.93	0.60	40.38
Menstrual changes						
NULL	–	–	–	–	–	–
Nightmares						
NULL	–	–	–	–	–	–
Rash						
NULL	–	–	–	–	–	–
Shortness of Breath						
No	REF	–	–	REF	–	–
Yes	**4.29**	**1.11**	**16.64**	4.62	0.88	24.12
Taste or smell changes						
No	1.01	0.31	3.26	REF	–	–
Yes	NULL	–	–	1.04	0.37	2.95
Tiredness or fatigue						
NULL						
Weight loss						
No	REF	–	–	REF	–	–
Yes	2.63	0.52	13.21	2.86	0.52	15.58
COVID symptoms lasting 30 days or more						
Anxiety						
No	REF	–	–	REF	–	–
Yes	15.97	0.44	574.67	19.70	0.45	860.56
Brain Fog						
NULL						
Chest Pain						
NULL						
Constipation						
NULL						
Depression						
No	REF	–	–	REF	–	–
Yes	4.00	0.55	29.09	4.07	0.39	42.35
Diarrhea						
No	REF	–	–	REF	–	–
Yes	6.96	0.26	184.49	7.45	0.28	198.12
Dizziness						
No	REF	–	–	REF	–	–
Yes	**14.00**	**1.80**	**108.80**	**14.55**	**1.73**	**122.37**
Exercise inability						
NULL						
Hair Loss						
NULL						
Headache						
NULL						
Heart palpitations						
NULL						
Hypersomnia						
No	REF	–	–	REF	–	–
Yes	**27.31**	**2.54**	**293.80**	**38.88**	**2.92**	**518.16**
Insomnia						
NULL	–	–	–	–	–	–
Joint or muscle pain						
No	REF	–	–	REF	–	–
Yes	**18.57**	**2.02**	**170.90**	**22.27**	**1.77**	**280.03**
Menstrual changes						
NULL						
Nightmares						
NULL						
Rash						
No	REF	–	–	REF	–	–
Yes	6.96	0.26	184.49	7.45	0.28	198.12
Shortness of breath						
No	REF	–	–	REF	–	–
Yes	**26.87**	**3.37**	**214.05**	**36.24**	**3.45**	**380.49**
Taste or smell changes						
NULL						
Tiredness or fatigue						
NULL						
Weight loss						
No	REF	–	–	REF	–	–
Yes	**30.42**	**3.69**	**251**	**38.97**	**3.42**	**444.67**

*Adjusted for age (except when age is in model).

**Adjusted for age and race (except when age or race is in model, where adjustment is for the other variable).

Bold = significant association at alpha = 0.05.

NULL = Model failed to converge.

**Table 10:** Multivariate analysis for the association between characteristics of population and long COVID adjusted sequentially for age, race and income and age, race, income and healthcare access.

Predictor	Odds Ratio	Adjusted***	95% CI (ub)	Odds Ratio	Adjusted****	95% CI (ub)
		95% CI (lb)			95% CI (lb)	
Severity of symptoms						
Mild	REF	–	–	NULL	–	–
Moderate	2.12	0.18	25.69	NULL	–	–
Severe	**29.58**	**1.38**	**632.53**	NULL	–	–
Race						
Hispanic	1.23	0.14	10.66	1.02	0.09	12.31
Non-Hispanic Black/African American	0.83	0.05	14.72	0.81	0.05	14.14
Non-Hispanic Asian, Native Hawaiian or Pacific Islander, or Other	1.59	0.07	35.55	1.79	0.07	44.51
Non-Hispanic White	REF			REF		
Annual HH income						
Less than $25,000	N/A	N/A	N/A	1.49	0.08	26.90
$25,000 to $49,000	N/A	N/A	N/A	0.63	0.01	30.46
$50,000 to $74,000	N/A	N/A	N/A	0.88	0.12	6.69
$75,000 or more	REF			REF		
Highest education level	NULL	–	–	NULL	–	–
High school or less	–	–	–	–	–	–
College or more	–	–	–	–	–	–
65 years and older					–	–
No	REF	–	–	REF	–	–
Yes	2.11	0.10	44.65	2.24	0.11	46.52
Health insurance coverage						
No	0.63	0.09	4.54	N/A	N/A	N/A
Yes	REF	–	–	REF	–	–
Heart disease						
No	REF	–	–	REF	–	–
Yes	**6.09**	**1.22**	**30.44**	**6.00**	**1.15**	**31.28**
Blood pressure						
No	REF	–	–	REF	–	–
Yes	1.92	0.47	7.94	2.14	0.52	8.79
Stroke						
No	REF	–	–	REF	–	–
Yes	1.37	0.04	48.86	1.40	0.05	37.69
Asthma						
No	REF	–	–	REF	–	–
Yes	5.94	0.87	40.34	5.98	0.84	42.67
Cancer						
No	REF	–	–	REF	–	–
Yes	1.37	0.25	7.53	1.35	0.22	8.13
High cholesterol						
No	REF	–	–	REF	–	–
Yes	5.28	0.87	32.10	5.26	0.63	43.66
Overweight or obesity						
No	REF			REF		
Yes	1.38	0.18	10.72	1.34	0.16	11.44
Diabetes or high blood sugar						
No	REF	–	–	REF	–	–
Yes	3.49	0.36	34.12	3.49	0.36	34.30
Poor mental health						
No	REF	–	–	REF	–	–
Yes	2.34	0.51	10.8	2.43	0.50	11.89
Poor physical health						
No	REF	–	–	REF	–	–
Yes	3.75	0.41	34.74	3.76	0.30	46.84
COPD						
No	REF	–	–	REF	–	–
Yes	6.28	0.23	175.05	6.29	0.19	206.72
Chronic kidney disease						
No	REF	–	–	REF	–	–
Yes	7.85	0.75	81.93	7.74	0.72	83.19
COVID symptoms anytime during infection						
Anxiety						
NULL						
Brain fog						
No	REF	–	–	REF	–	–
Yes	2.29	0.33	15.79	2.20	0.28	17.54
Chest pain						
No	REF	–	–	REF	–	–
Yes	**7.21**	**1.23**	**42.13**	**7.15**	**1.13**	**45.23**
Constipation						
NULL						
Depression						
NULL						
Diarrhea						
No	REF	–	–	REF	–	–
Yes	0.68	0.10	4.42	0.64	0.12	3.49
Dizziness						
No	REF	–	–	REF	–	–
Yes	2.97	0.55	16.16	2.84	0.47	17.01
Exercise Inability	REF	–	–	REF	–	–
No	2.84	0.46	17.53	2.93	0.42	20.26
Headache						
No	REF			REF		
Yes	1.06	0.24	4.78	1.08	0.24	4.93
Heart palpitations						
No	REF			REF		
Yes	**6.68**	**1.06**	**42.07**	**6.59**	**1.08**	**40.18**
Hypersomnia						
NULL	–	–	–	–	–	–
Insomnia						
No	REF	–	–	REF	–	–
Yes	8.31	0.23	296.5	9.22	0.11	760
Joint or muscle pain						
No	REF	–	–	REF	–	–
Yes	5.93	0.27	128.21	5.80	0.25	135.45
Menstrual changes						
NULL						
Nightmares						
NULL						
Rash						
NULL						
Shortness of breath						
No	REF	–	–	REF	–	–
Yes	**5.02**	**1.24**	**20.30**	**4.97**	**1.16**	**21.36**
Taste or smell changes				REF		
No	REF			REF		
Yes	0.58	0.19	1.79	0.58	0.188	1.79
Tiredness or fatigue						
NULL						
COVID symptoms lasting 30 days or more						
Anxiety						
NULL	–	–	–	–	–	–
Brain fog						
NULL	–	–	–	–	–	–
Chest pain						
NULL	–	–	–	–	–	–
Constipation						
NULL	–	–	–	–	–	–
Depression						
No	REF	–	–	REF	–	–
Yes	1.70	0.07	42.88	1.58	0.09	28.95
Diarrhea						
No	REF	–	–	REF	–	–
Yes	14.96	0.34	665.41	14.67	0.22	964.66
Dizziness						
No	REF	–	–	REF	–	–
Yes	**26.12**	**1.45**	**471.62**	27.17	0.83	889.87
Exercise inability						
NULL	–	–	–	–	–	–
Hair Loss						
NULL	–	–	–	–	–	–
Headache						
NULL	–	–	–	–	–	–
Hair Loss						
NULL	–	–	–	–	–	–
Headache						
NULL						
Heart palpitations						
NULL	–	–	–	–	–	–
Hypersomnia						
No	REF	–	–	REF	–	–
Yes	24.18	0.87	669.90	22.92	0.54	971.08
Insomnia						
NULL	–	–	–	–	–	–
Joint or muscle pain						
No	REF	–	–	REF	–	–
Yes	**35.40**	**2.84**	**442.04**	NULL		
Menstrual changes						
NULL	–	–	–	–	–	–
Nightmares						
NULL	–	–	–	–	–	–
Rash						
No	REF	–	–	REF	–	–
Yes	14.96	0.34	665.41	14.67	0.22	964.66
Shortness of breath						
NULL	–	–	–	–	–	–
Taste or smell changes						
NULL	–	–	–	–	–	–
Tiredness or fatigue						
NULL	–	–	–	–	–	–
Weight loss						
No	REF	–	–	REF	–	–
Yes	**26.12**	**1.45**	**471.62**	27.17	0.83	889.87

***Adjusted for age, race, and income (except when age, race, or income are in model, where adjustment is for other 2 variables).

****Adjusted for age, race, income, and health insurance coverage (except when age, race, income, and health insurance coverage are in model, where adjustment is for other 3 variables).

Bold = significant association at alpha = 0.05.

NULL = Model failed to converge.

REF = reference group.

**Figure 6:**
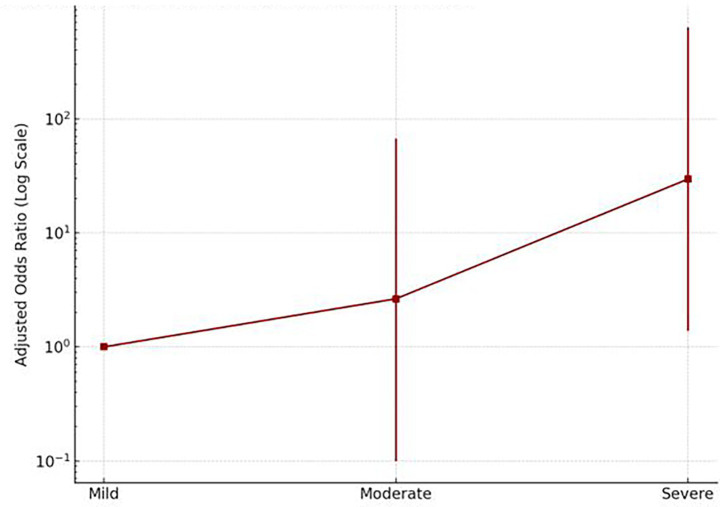
Adjusted odds of long COVID by acute symptom severity.

After adjusting for age; age and race; and age, race, and income, individuals who experienced severe acute COVID-19 symptoms had nearly 30 times higher odds of long COVID (OR = 29.58; 95% CI: 1.38–632.53) compared to those with mild symptoms. Even moderate symptoms were associated with elevated odds (OR = 2.64) (Figure 6). This strong association suggests that severe acute illness is a notable predictor of long COVID. In contrast, no statistically significant association was observed between mild symptoms and the development of long COVID.

After adjustment for covariates, individuals who reported heart disease had significantly higher odds of developing long COVID compared to those without heart disease (OR = 6.00, 95% CI [1.15, 31.28]). Although asthma was significantly associated with long COVID in the unadjusted (crude) model, the association was attenuated and no longer statistically significant after adjustment (OR = 3.49, 95% CI [1.05, 11.59]). Poor physical health remained a significant predictor of long COVID after adjusting for age and race, with affected individuals exhibiting 4.20 times higher odds of developing the condition compared to those reporting good physical health (OR = 4.20, 95% CI [1.12, 15.75]). These findings underscore the importance of preexisting cardiovascular and general health conditions in the risk profile for long COVID.

The association between symptoms experienced at any time during infection and symptoms experiences thirty days or more after infection and long COVID was also explored. This provided additional valuable insight into the conditions that may exacerbate the development of long COVID. Those who experienced anxiety at any time during their infection were 22.94 times higher odds developing long COVID after adjusting for age and race (OR = 22.94, 95% CI [2.01, 262.31]) and those who experienced chest pain at any time during their infection were at increased odds of developing long COVID after adjusting for age, race, income, and health insurance coverage (OR = 7.15, 95% CI [1.13, 45.23]) in comparison to those who did not experience those symptoms during infection. Those who experienced constipation at any time during their infection were 16.81 higher odds of developing long COVID in comparison to those who did not experience constipation (OR = 16.81, 95% CI [1.33, 213.23]). Those who experienced heart palpitations (OR = 6.59, 95% CI [1.08, 40.18]) and shortness of breath (OR = 4.97, 95% CI [1.16, 21.36]) were 6.59 times higher odds of developing long COVID after adjustments for age, race, income, and access to healthcare.

By contrast, no statistically significant associations were observed between long COVID and sociodemographic factors such as race, education, income, or access to healthcare in either the crude or adjusted models.

## 4 Discussion

This study contributes important insights into the prevalence, characteristics, and risk factors of post-acute sequelae of COVID-19 (PASC), also known as long COVID, among a community-based population in Third Ward, Houston. Notably, it highlights the significant associations between symptom severity during acute infection and specific comorbidities and symptoms with the likelihood of developing long COVID. Specifically, severe symptoms during initial infection, comorbid conditions such as heart disease and asthma, and physical and psychological symptoms like poor physical health, anxiety, chest pain, constipation, heart palpitations, hypersomnia, and shortness of breath were associated with increased likelihood of long COVID. Conversely, socio-demographic characteristics such as race, income, education level, and access to healthcare did not show significant associations with the development of long COVID in adjusted models. These findings emphasize that the manifestation of long COVID is more intricately linked to clinical health experiences than to socio-economic disparities, although the latter may still play an indirect role in healthcare access and disease management.

### 4.1 Acute symptom severity

The relationship between severe acute COVID-19 and subsequent long COVID is affirmed by the findings. Individuals who experienced severe symptoms had nearly 30 times higher odds of developing long COVID compared to those with mild symptoms, consistent with prior research (1, 22). These results align with findings from the NIH RECOVER Initiative, which reported an increased risk of PASC among individuals with more severe initial illness, especially those requiring hospitalization or mechanical ventilation (22). Severe acute illness may trigger prolonged inflammatory responses or tissue damage, contributing to persistent symptoms.

### 4.2 Comorbidities

The significant association of heart disease and asthma with long COVID adds to existing literature recognizing chronic comorbidities as risk enhancers (23, 24). In this study, participants with heart disease had six times the odds of developing long COVID compared to those without. Although asthma lost statistical significance after full adjustment, it was notable in crude models. This aligns with evidence from Hill et al. (22), who found chronic lung disease to be one of the strongest predictors of PASC. Moreover, the study found that poor physical health significantly increased the odds of long COVID, consistent with research by Mandal et al. (13) and Houben-Wilke et al. (25), who identified fatigue, breathlessness, and poor functional recovery as common post-COVID conditions.

### 4.3 Symptom clusters

The presence of specific acute symptoms, particularly anxiety, chest pain, heart palpitations, constipation, hypersomnia, and shortness of breath, demonstrated strong associations with long COVID. This mirrors the symptom clusters identified by the FAIR Health (10) report, which found that fatigue, breathing abnormalities, and persistent cough were among the most commonly co-occurring symptoms across age and gender groups. Our findings also corroborate those of McCorkell et al. (26), whose comprehensive patient-led study emphasized fatigue, cognitive dysfunction, and post-exertional malaise as dominant features of long COVID persisting for months. The high odds associated with hypersomnia and anxiety in this study further support calls for integrated physical and mental healthcare in managing long COVID.

### 4.4 Sociodemographic factors and health equity

Interestingly, while this study included a racially diverse sample with a majority of African American respondents, race and ethnicity were not significantly associated with long COVID. This finding diverges from some national-level studies, such as those by Jacobs et al. (27) and Hill et al. (22), which documented underdiagnosis of PASC in minority populations, particularly Black Americans. However, it supports Louie and Wu (28), who found that socioeconomic factors modulated the relationship between race and long COVID, with effects appearing buffered in lower-income communities. In our sample, the homogeneity of healthcare access limitations and high comorbidity prevalence in the Third Ward may have attenuated detectable racial differences. Further research is warranted to explore how structural inequities and community-level factors influence long COVID trajectories.

### 4.5 Healthcare access and economic factors

Another important finding is the absence of significant associations between long COVID and educational attainment, income, and healthcare access. While surprising, this may reflect limitations in the survey design or the complexity of long COVID, where individual biological and clinical experiences outweigh traditional socio-demographic predictors. Notably, over 78% of households in the study lacked health insurance, and 16% reported difficulty accessing care, often due to financial barriers. These structural constraints may have affected both the reporting and experience of long COVID, contributing to potential underdiagnosis or delayed treatment.

### 4.6 Vaccination data and viral strains

The role of vaccination was not directly addressed in this study, which is a limitation. Our study occurred at a time when testing and vaccination coverage varied widely, thus the instrument utilized did not capture vaccine status. Research from Antonelli et al. (29) demonstrated that vaccinated individuals had reduced odds of developing long COVID, especially after the second dose, and experienced fewer symptoms if infected. Given that most infections reported by participants occurred 12 months or more before the survey, many may have been exposed before widespread vaccine availability. Additionally, inability to clinically verify vaccination and immunization data contributed to its exclusion from the survey instrument. This limits the ability to contextualize the findings within the shifting epidemiological landscape, including the rise of variants like Delta and Omicron, which have shown different patterns of transmissibility and severity.

Vaccination against SARS-CoV-2 has been associated with a reduction in the risk of developing long COVID, although the magnitude of this effect varies depending on the study design and operational definitions used. Several studies suggest a protective role, but results are nuanced. Antonelli et al. (29) based on app-reported symptoms, observed that individuals who received two vaccine doses had approximately half the risk of experiencing symptoms persisting beyond 4 weeks following infection. However, this study’s reliance on self-reported data and a relatively short follow-up period may limit generalizability to definitions of long COVID that require longer symptom duration.

Other studies using broader or more clinically validated definitions have reported stronger effects. For instance, a population-based cohort analysis by Krishna et al. (30) found a 79% reduction in hospital admission for post-acute sequelae 6 months after infection among vaccinated individuals in the United Kingdom. Similarly, Ayoubkhani et al. (31) analyzed data from a UK community-based cohort and found that vaccination after SARS-CoV-2 infection was associated with a 12.8% reduction after the first dose and an 8.8% reduction after the second dose. Population-level registry studies have also supported a protective association. For example, Ayoubkhani et al. (32) found that individuals vaccinated with two doses had signifigantly lower odds of reporting long COVID. Conversely, other large-scale electronic health record (EHR) studies, such as Al-Aly et al. (33) found only modest reductions in long COVID risk among vaccinated individuals. This discrepancy may be due in part to the older population studied, the inclusion of pre-existing comorbidities such as cardiovascular disease, and broader outcome definitions encompassing systemic post-viral sequelae.

Given the heterogeneity in study findings, our study acknowledges the importance of incorporating vaccination status into long COVID assessments. Although our data did not initially include this variable, its significance as a potential confounder or effect modifier is noted, and we recommend that future studies incorporate vaccination history. Despite the lack of vaccination data, the timing of infections in the study provides valuable insights. Most participants experienced symptoms more than a year before the survey, indicating that long COVID persisted over time. The transition from early variants to Delta and Omicron introduced changes in symptom profiles, with Omicron associated with higher transmissibility but lower severity (29). However, this study could not stratify findings by variant or time since infection, underscoring the need for more granular data on timing and viral strain in future studies to enhance data accuracy and interpretability of findings.

### 4.7 Multisystemic impact and clinical implications

The multisystemic nature of symptoms reported—ranging from neurological and gastrointestinal to cardiovascular—confirms the wide-ranging impact of long COVID observed in other studies (1, 34). For instance, this study’s findings of persistent constipation and chest pain reflect gastrointestinal and cardiac involvement, respectively, while symptoms such as brain fog, hypersomnia, and anxiety indicate neurological and psychological dimensions. Studies by Blackett et al. (35) and Xu et al. (36) reinforce this, highlighting gastrointestinal and neurological sequelae as part of the long COVID syndrome. These varied symptom clusters necessitate multidisciplinary management approaches that include mental health, cardiology, pulmonology, and rehabilitation services.

### 4.8 Mental health burden

Psychological symptoms, particularly anxiety and depression, also emerged as prevalent among respondents. This aligns with findings from Houben-Wilke et al. (25), who reported high levels of anxiety, depression, and PTSD symptoms in patients with persistent complaints at 3 and 6 months. In the current study, anxiety showed one of the strongest associations with long COVID, indicating a need to integrate behavioral health services into post-COVID care models. Similarly, the findings resonate with Ladds et al. (12), whose qualitative research underscored the emotional toll and difficulties patients faced navigating healthcare services during recovery.

### 4.9 Gastrointestinal symptoms

Notably, gastrointestinal symptoms, such as constipation, were significantly associated with long COVID, confirming emerging literature that documents persistent GI symptoms even months after infection (35). While often overlooked in clinical assessments, GI manifestations can severely affect quality of life and may be linked to viral persistence or gut microbiome disruption (34). These findings advocate for including gastrointestinal evaluation in long COVID protocols.

### 4.10 Interpretation and public health relevance

The study highlights a complex interplay of acute symptom severity, comorbidities, and individual symptom experiences in predicting long COVID. The absence of racial, economic, and educational associations does not negate the role of structural inequities but rather points to the dominant influence of biological and clinical risk factors in this specific population. However, systemic inequities may still modulate who gets diagnosed, treated, and included in research. The lack of significant findings for diabetes contrasts with studies such as Assad et al. (23) and Fernández-de-las-Peñas et al. (37), which found associations between diabetes and long COVID. The absence of association in our study may be due to limitations in sample size, lack of detailed glycemic control data, or variability in self-reporting. Furthermore, the comorbidity interplay, such as between hypertension and diabetes, may obscure individual associations without detailed stratification or interaction modeling.

These findings have several public health implications. First, they underscore the need for symptom-based screening tools that prioritize clinical severity and comorbid history over demographic profiling. Second, they call for community-based education and support services, especially in historically underserved neighborhoods like the Third Ward, where distrust of the healthcare system may delay care-seeking (38). Third, they emphasize the urgency of integrated care pathways that can address the multisystem nature of long COVID.

Despite its strengths, this study has limitations that warrant consideration. Data were based on self-reported surveys, introducing recall bias and potential misclassification of respondent. The reliance on one household member to report for the entire household may lead to inaccuracies in symptom recall or comorbidity reporting. Additionally, the cross-sectional design precludes causality assessments and limits understanding of symptom progression. With respect to recovery data, our survey did not include longitudinal follow-up or specific questions regarding symptom resolution or duration. Participants were asked to report their current and past symptoms at the time of the survey, but without a time-course element, we were unable to systematically assess recovery trajectories. We plan to incorporate these elements into future studies. The absence of vaccine status, testing date, and viral strain data further restricts interpretations. Moreover, missing data and limited sample size may have underpowered the detection of some associations or contributed to unstable odds ratios. While the use of weighted data and cluster-based sampling enhances generalizability, the relatively small number of directly surveyed households (*N* = 168) may limit statistical precision, as reflected in some wide confidence intervals. Larger studies are needed to validate and strengthen these estimates. We also acknowledge that the lack of a comparison group limits the interpretability of symptom frequency and severity. Future studies should consider including a comparison group to enhance contextual understanding of the findings. Lastly, although the survey instrument included open-ended items and general questions about the impact of COVID-19 on daily life, a validated scale for functional assessment was not utilized. Future work should incorporate standardized instruments to more accurately assess functional outcomes. Stratified analyses by sex, age, and time since infection are also recommended to improve comparability and analytic precision.

Nevertheless, this study contributes valuable insights to the evolving understanding of long COVID, particularly within a racially diverse and underrepresented urban population. Future research should adopt longitudinal designs, integrate clinical validation of symptoms, and examine variant-specific effects, vaccine influence, and the impact of evolving social determinants. Such work is essential for informing equitable, effective, and patient-centered responses to the long-term aftermath of COVID-19.

## 5 Conclusion

Post-COVID conditions, also known by such terms as long COVID and post-acute sequelae of COVID-19, have become an issue of growing national concern (10). COVID-19 is a multisystemic disease with long-term impact on almost all body systems. This study of patients who were diagnosed with post-acute sequelae of COVID-19 resulted in several notable findings that are important for individuals diagnosed with COVID-19, medical providers, researchers, and policymakers. Based upon the associations between comorbid conditions and long COVID, addressing baseline health before another pandemic arises is critical. Moreover, clinical multi-disciplinary evaluation must be conducted to manage and minimize the effect of long-term COVID-19 and follow-up of symptoms post-COVID period as persistent symptoms disrupt quality of life (3). Patients must also be educated on the long-term effects of COVID-19 in an effort to not only treat but prevent COVID-19 infection altogether.

Minority populations must also be more meaningfully engaged in research, however, if we are to engage minority populations more fully in research studies on long COVID and COVID-19 clinical trials, we must improve relationships between patients and providers, which may mean acknowledging implicit bias to eliminate it. This requires acknowledgment of the mistrust in the healthcare system and its impacts on the provider-patient relationship.

The final recommendations involve policies that fund centers that conduct research and educate clinicians to promote collaboration between the research and the medical community. Additionally, pandemics, viruses, and infectious diseases must be integrated into curriculums not to replicate the miscues observed during the COVID-19 pandemic. Lastly, health care should involve a multidisciplinary approach to address the patient as a whole in the post-COVID period and further research into the underlying mechanisms of COVID-19 is needed to better understand and alter the course of disease.

## Data Availability

The raw data supporting the conclusions of this article will be made available by the authors, without undue reservation.
